# Examination of the health and safety aspects of 28-days ingestion of a supplement containing slow-release caffeine

**DOI:** 10.1186/1550-2783-11-S1-P17

**Published:** 2014-12-01

**Authors:** Adam J Wells, Jay R Hoffman, Adam M Gonzalez, Kyle S Beyer, Adam R Jajtner, Jeremy R Townsend, Michael LaMonica, Ameilia Miramonti, Mattan W Hoffman, Leonardo P Oliveira, David H Fukuda, Maren S Fragala, Jeffrey R Stout

**Affiliations:** 1 University of Central Florida, Orlando, FL, USA

## Background

Moderate amounts of caffeine can lead to an increase in both physical and mental task performance. However, a single dose of caffeine typically induces only 90-120 minutes of increased alertness and is often associated with an acute “crash” state following its metabolism. Recently, slow/sustained release caffeine (SRC) alternatives have been developed to prolong the effects of caffeine. While prior investigations have demonstrated the efficacy of SRC, there is currently limited information regarding the safety of SRC during prolonged usage. Therefore, the purpose of this study was to investigate the effects of 28-days of daily SRC ingestion on blood lipid profiles, comprehensive blood chemistry, and complete blood counts in young, healthy men and women.

## Methods

Forty healthy individuals (20 males, 20 females; age: 22.73 ± 3.06 years; height: 171.68 ± 10.45 cm; mass: 74.49 ± 15.51 kg; BMI: 25.08 ± 3.66 (kg/m2) who were regular consumers of caffeine volunteered to participate in this randomized, double-blind, placebo controlled study. While enrolled in the study, participants were permitted to maintain their normal caffeine intake. Following a 12-hour fast, participants reported to the Human Performance Laboratory (HPL) for pre-testing. Testing consisted of resting heart rate (RHR) and blood pressure (BP) measures, followed by a resting blood draw obtained from an antecubital vein in the superficial forearm using a 21-gauge disposable needle stick. Upon completion of pre-testing, participants supplemented with either Energize™ (SUPP) or placebo (PL) for 28 days. Daily supplementation was witnessed by lab personnel. On weekends, participants were provided 2 dosages of SUPP or PL to consume on each weekend day. Weekend supplementation was provided in zip lock bags, which were returned to demonstrate adherence. Post-testing occurred 24-hours after ingestion of the final dose and consisted of the same protocol at the same time of day as pre-testing. Within 24-hours of pre- and post-testing, blood samples were packaged along with a requisition form for analysis at a commercial laboratory. Statistical analysis of the data was accomplished using a 2 by 2 repeated measures analysis of variance (ANOVA) to determine between groups differences (SUPP vs. PL). Consent to publish the results was obtained from all participants.

## Results

Compliance was 98.3%. No significant differences between the groups were observed for resting cardiovascular measures, blood lipids, or complete blood counts. A significant difference between groups was observed for plasma glucose concentrations (p = 0.028); however, follow-up testing revealed that pre- to post-supplementation changes were not significant for either SUPP (p = 0.077) or PL (p = 0.116). No other between group differences were observed for metabolic blood chemistry. All variables remained within normal adult reference ranges. Study participants reported no adverse events during supplementation with either SUPP or PL.

## Conclusion

These findings indicate that 28 consecutive days of moderate SRC ingestion in caffeine users is both safe and tolerable. This may have important relevance in professions, where continuous operations demand peak cognitive and physical performance for sustained periods of time.

